# Synthesis and Evaluation of a Hybrid Miltefosine-Silver Nanoparticle Complex: Synergistic Interaction with Benznidazole Against *Trypanosoma cruzi*

**DOI:** 10.1007/s11686-025-01074-3

**Published:** 2025-06-12

**Authors:** Yener Özel, İbrahim Çavuş, Feyzullah Tokay, Sema Bağdat, Ahmet Özbilgin

**Affiliations:** 1 Department of Medical Microbiology, Balıkesir University Medical Faculty, Balikesir, Türkiye; 2 Department of Medical Parasitology, Manisa Celal Bayar University Medical Faculty, Manisa, Türkiye; 3 Faculty of Arts and Sciences, Department of Analytical Chemistry, Balikesir University, Balikesir, Türkiye

**Keywords:** Silver nanoparticles, Miltefosine, Synergy, *T. cruzi*

## Abstract

**Objective:**

Chagas disease is an infectious disease classified under neglected tropical diseases and caused by the protozoan parasite *Trypanosoma cruzi*. This study aimed to investigate the cytotoxic activity, antitrypanosomal efficacy, and combination effects with benznidazole of hybrid silver nanoparticles (AgNPs) synthesized with miltefosine against *T. cruzi* epimastigotes.

**Methods:**

In this study, a hybrid miltefosine (Mil)-silver nanoparticle (OA-MilAg-NP) complex was synthesized. The nanoparticles were characterized using FT-IR spectroscopy, transmission electron microscopy (TEM), and scanning electron microscopy (SEM) analyses. The cytotoxicity of the nanoparticles was assessed in L929 fibroblast cells, while their antitrypanosomal activity was evaluated against a *Trypanosoma cruzi* ATCC 50828 strain using the broth microdilution method. The interaction between the nanoparticle complex or miltefosine and benznidazole was analyzed using the checkerboard method.

**Results:**

FT-IR analysis demonstrated that the amylose surface was successfully coated with silver and miltefosine, confirming the successful synthesis of the hybrid complex. SEM analysis revealed that the nanoparticles exhibited a spherical morphology with varying sizes, while TEM analysis determined their sizes ranged between 10.14 and 18.42 nm. The OA-MilAg-NP complex exhibited high antitrypanosomal activity and a selectivity index twice as high as that of miltefosine. Synergistic interactions were observed in the combinations of the OA-MilAg-NP complex or miltefosine with benznidazole.

**Conclusion:**

The development of novel bioactive compounds with lower toxicity compared to traditional drugs has become essential for the treatment of Chagas disease. Drug repurposing combined with nanotechnology applications holds significant potential for improving therapeutic outcomes. The hybridization of miltefosine with silver nanoparticles, demonstrating strong antitrypanosomal activity and synergistic effects with benznidazole, may fill critical gaps in the literature.

**Graphical Abstract:**

In this study, oxidized amylose-miltefosine-silver (OA-MilAg-NP) hybrid nanoparticles were synthesized. The characterization of the nanoparticles was performed using FTIR, SEM, and TEM analyses. The cytotoxicity of the hybrid OA-MilAg-NPs was evaluated on the L929 fibroblast cell line. The antitrypanosomal activity of the synthesized nanoparticles against *T. cruzi* isolates was determined using the in vitro broth microdilution method, while their synergy with benznidazole was assessed using the checkerboard method. 
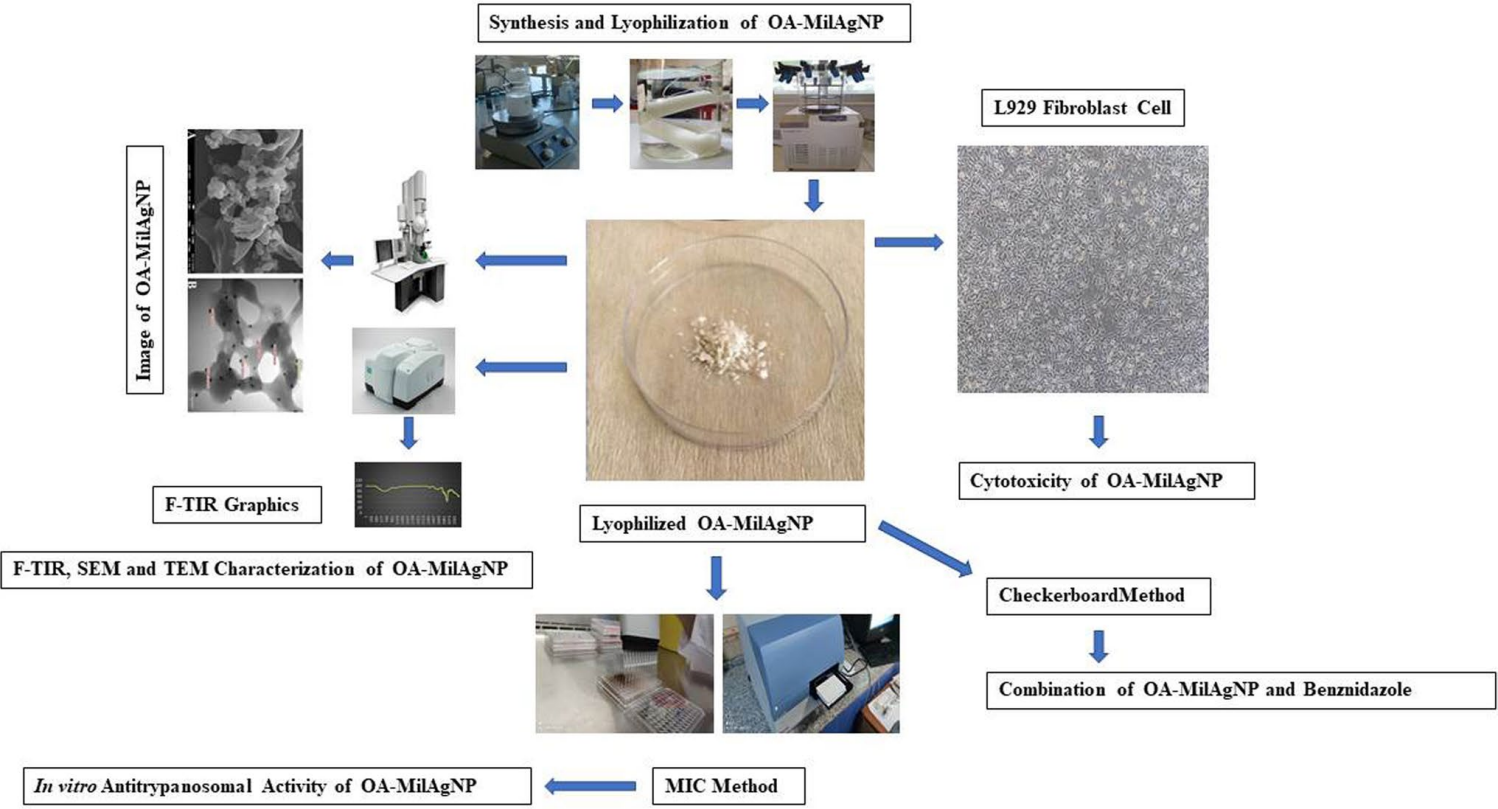

## Introduction

Chagas disease is an infectious disease categorized under neglected tropical diseases, caused by the protozoan parasite *Trypanosoma cruzi*. The infection can be transmitted through Triatomine bugs (vector-borne), oral routes (foodborne), during pregnancy or childbirth (congenital), blood or blood products, organ transplantation, and laboratory accidents. It is estimated that approximately 6–7 million people worldwide are infected with *T. cruzi*, and the disease causes around 12,000 deaths annually. Despite its increasing global presence, Chagas disease is primarily observed in the endemic regions of 21 Latin American countries, where transmission largely depends on the presence of vectors. Currently, it is estimated that approximately 75 million people are at risk of infection [1]. The high levels of global human mobility due to migration, natural disasters, war, and tourism may lead to the emergence of neglected tropical diseases like Chagas disease in non-endemic regions as well [2].

Most acute infections are asymptomatic or present with non-specific symptoms such as fever, lymphadenopathy, and hepatosplenomegaly. Regardless of the mode of transmission, severe manifestations include myocarditis and encephalitis, with the severity of the disease likely associated with the parasitic load. Most acute infections go unrecognized and are rarely diagnosed. Approximately 30% of chronically infected patients develop Chagas cardiomyopathy years later. Chagas cardiomyopathy can lead to stroke, heart failure, thromboembolic events, and death [3].

Chagas disease, caused by *Trypanosoma cruzi*, is treated with two drugs: benznidazole and nifurtimox. However, these drugs have several disadvantages, including their effectiveness being limited to the acute or early stages of infection, the occurrence of side effects during treatment, and the parasite developing resistance to their efficacy. Therefore, it is essential to identify new, safe, and effective therapeutic alternatives for the treatment of Chagas disease. Scientists are working on several strategies to address this issue. These include modifying traditional drug dosages, redesigning drugs, combination therapies, and nanotechnology [4]. Some studies in the literature report remarkable results regarding inorganic nanoparticles with antimicrobial properties, such as gold, silver, zinc oxide, and titanium dioxide nanoparticles [5, 6].

Nanotechnology is a scientific discipline based on the synthesis and utilization of particles at the nanometer scale for various purposes. The physicochemical and biological properties of nanoparticles (NPs) vary depending on their morphological and structural characteristics, particle size, and surface area [7]. Recently, scientists have focused extensively on research related to nanotechnology applications. Nanotechnology is employed in the execution of precise medical procedures and contributes to fields such as diagnostics, disease treatment, regenerative medicine, gene therapy, dentistry, oncology, the cosmetic industry, drug delivery, and therapeutics [8].

In our previous study, we observed that hybrid curcumin-silver nanoparticles exhibited strong efficacy and low cytotoxicity against *Leishmania* species, which are closely related to *T. cruzi* [5]. These findings form the basis of the hypothesis that miltefosine—an orally administrable drug with limited treatment alternatives for Chagas disease—could similarly demonstrate high efficacy and low cytotoxicity when synthesized as a hybrid nanoparticle complex.

Although benznidazole remains the primary treatment option for Chagas disease, its clinical use is hindered by adverse effects, variable efficacy in chronic stages, and the emergence of drug resistance. These limitations emphasize the need for new therapeutic strategies that are both safer and more effective. In this context, our study proposes a novel nanotechnology-based approach by developing a hybrid silver nanoparticle complex incorporating miltefosine, a repurposed antileishmanial drug with established oral bioavailability. This innovative formulation is designed to enhance antitrypanosomal efficacy while potentially reducing host toxicity. Furthermore, by evaluating its combination with benznidazole, the study aims to explore synergistic effects that could improve treatment outcomes and help overcome existing therapeutic barriers. This study reports for the first time the synthesis and antitrypanosomal evaluation of a novel hybrid complex composed of miltefosine and silver nanoparticles, with the goal of enhancing therapeutic efficacy and reducing toxicity.

## Material And Methods

### Ethical Approval

No human or animal materials were used in this study. The experiments were conducted using parasite strains preserved in liquid nitrogen. Therefore, ethical approval is not required.

### Trypanosoma cruzi Strain and Cell Line

The *Trypanosoma cruzi* ATCC 50825 reference strain and the L929 mouse fibroblast cell line (American Type Culture Collection, USA) used in this study were obtained from the Parasite Bank of Manisa Celal Bayar University Faculty of Medicine.

### Drugs

Miltefosine (B2693-072166) was obtained from BOCScience (USA), and benznidazole (419,656) was obtained from Sigma-Aldrich (USA).

### Synthesis of Nanoparticle Complexes

#### Synthesis of Oxidized Amylose

15 mL of 30% H₂O₂, 2 mL of 0.05% CuSO₄, and 60 g of amylose were combined and stirred at 40 °C for 15 min. At the end of this period, 400 mL of boiling water was added to the mixture, which was then stirred at 75 °C for an additional 15 min. After this step, another 400 mL of boiling water was added, and the mixture was stirred at 100 °C for 30 min. The solution was cooled to room temperature and centrifuged at 3000 rpm for 20 min. To remove copper ions from the resulting supernatant, dialysis was performed. The dialysis bag was dialyzed against distilled water at room temperature for two days, protected from light [9].

#### Synthesis of Hybrid Oxidized Amylose Miltefosine Silver Nanoparticle (OA-MilAg-NP) Complex

To the dialyzed oxidized amylose supernatant, 100 mL of boiling water and 75 mL of 0.02 mol/L AgNO₃ were added and kept at 100 °C in a dark environment for 120 min. Subsequently, 1 g of miltefosine was added to the mixture, which was cooled to room temperature with constant stirring. The mixture was centrifuged at 12,000 rpm for 15 min to remove excess miltefosine. Similarly, the excess AgNO₃ in the mixture was removed by dialysis [10]. The synthesized hybrid nanoparticle complex was lyophilized using the Christ Alpha 1–2 LD Plus lyophilization device at the Balikesir University Science and Technology Application and Research Center.

#### FT-IR, SEM, and TEM Characterization of OA-MilAg-NP Complex

The FT-IR spectra of the hybrid nanoparticle complexes were recorded using a Perkin Elmer Spectrum 65 model device with a wavenumber range of 4000 cm⁻^1^ to 400 cm⁻^1^, located at Balikesir University, the Department of Analytical Chemistry, Faculty of Art and Science. Scanning electron microscopy (SEM) images were obtained using the JEOL JSM-7100F model device, and transmission electron microscopy (TEM) images were acquired using the JEOL JEM-1400 Plus model device, both located at the Çanakkale Onsekiz Mart University, Science and Technology Application and Research Center.

#### Preparation of OA-MilAg-NP Complex, Miltefosine, and Benznidazole Stock Solutions

The hybrid nanoparticle stock solution was prepared at a concentration of 10,000 μg/mL in sterile distilled water. Benznidazole stock was prepared at 12,400 μg/mL in ethanol, and a 1:10 dilution with distilled water was performed to obtain a working concentration of 1,240 μg/mL. Miltefosine was prepared directly as a working solution at a concentration of 1,024 μg/mL using sterile distilled water.

#### Determination of Cytotoxic Activity of OA-MilAg-NP Complex, Miltefosine, and Benznidazole

Fibroblasts preserved in liquid nitrogen were treated with RPMI-1640 and passaged into RPMI-1640 medium containing 10% FBS (fetal bovine serum). The cells were incubated at 37 °C with 5% CO₂ for 48 h. The number and viability of proliferating cells were determined using Trypan Blue staining and a hemocytometer. A 100 µL cell suspension containing 10^5^ cells/mL was distributed into 96-well microplates. Serial dilutions of the OA-MilAg-NP suspension (final concentration range: 5000–39 µg/mL), miltefosine (final concentration range: 512–0.125 µg/mL), and benznidazole (final concentration range: 640–0.60 µg/mL) were prepared in a separate microplate and transferred to the microplates containing the cells. The plates were incubated at 37 °C with 5% CO₂ for 48 h. Cell viability was determined using the MTT (3-(4,5-dimethylthiazol-2-yl)-2,5-diphenyltetrazolium bromide) assay [11], and absorbance values were measured at 570 nm using a Thermo Varioskan model spectrophotometer (USA). IC_50_ values indicating cytotoxic activity were calculated using GraphPad Prism 8.4.2 software. The cytotoxic activity tests were repeated three times on different days [7].

#### Determination of Antitrypanosomal Activity of OA-MilAg-NP Complex, Miltefosine, and Benznidazole

The *Trypanosoma cruzi* ATCC 50828 strain, thawed from liquid nitrogen, was cultured in LIT and NNN medium [12] and brought to the logarithmic phase in RPMI-1640 medium containing 10% FBS. For the determination of antitrypanosomal activity, 100 μL of RPMI-1640 medium was distributed into each well of sterile, flat-bottom, 96-well microplates. Serial dilutions of the hybrid NP complex were prepared in the range of 2500–19.5 μg/mL, miltefosine in the range of 128–1 μg/mL, and benznidazole in the range of 320–0.30 μg/mL. A 100 μL epimastigote suspension at a concentration of 1 × 10^5^/mL was added to all wells except the negative control, and the plates were incubated at 26 ± 1 °C for 24, 48, and 72 h [13]. The morphological structure and motility of the epimastigotes in all wells were observed under an inverted microscope. The lowest concentration of the active substance at which all parasites were immobile and their morphology was disrupted was determined as the minimum parasiticidal concentration (MPC). Epimastigote viability was assessed using the MTT (3-(4,5-dimethylthiazol-2-yl)-2,5-diphenyltetrazolium bromide) assay [11]. Absorbance values were measured at 570 nm using a Thermo Varioskan model spectrophotometer (USA). IC_50_ values indicating antitrypanosomal activity were calculated using GraphPad Prism 8.4.2 software. Efficacy tests were repeated three times on different days [7].

#### Determination of Selectivity Index (SI) Values for OA-MilAg-NP Complex, Miltefosine, and Benznidazole

The SI value was calculated using the following formula by dividing the IC_50_ value obtained for fibroblast cells (L929) by the IC_50_ value obtained for *T. cruzi* epimastigotes:

SI = IC_50_ value for fibroblasts / IC_50_ value for *T. cruzi* epimastigotes.

SI values greater than 10.00 indicate that the tested substance exhibits higher selectivity against epimastigotes than fibroblasts [14].

#### Determination of Interactions Between OA-MilAg-NP Complex and Miltefosine, with Benznidazole

The interaction between the OA-MilAg-NP complex and miltefosine with benznidazole was determined using the checkerboard method. Two 96-well microplates were used for each combination. In the first microplate, 100 μL of RPMI-1640 medium was distributed into all wells. Serial dilutions of the OA-MilAg-NP complex and miltefosine were performed vertically, starting from three dilutions above their IC_50_ values and proceeding five dilutions below. In the second microplate, 110 μL of RPMI-1640 medium was distributed into all wells up to the 8th column. A 110 μL solution of benznidazole was added to the wells in the 8th column, and horizontal serial dilutions were performed from right to left. The dilutions in the second microplate were then transferred to the corresponding wells in the first microplate in 100 μL volumes [15]. In all wells of the first microplate, except for the medium and sterility control wells, 100 μL of a parasite suspension at a concentration of 10^5^ epimastigotes/mL was added. Four wells were used for each control: growth control (RPMI-1640 + epimastigotes), medium control (RPMI-1640), and sterility control (RPMI-1640 + hybrid NP complex/miltefosine/benznidazole). The microplates were incubated at 26 °C for 24, 48, and 72 h. The morphological structure and motility of the epimastigotes in all wells were observed under an inverted microscope. The lowest concentration of the active substance at which all parasites were immobile and their morphology disrupted was determined as the minimum parasiticidal concentration (MPC) [7].

The interaction between the OA-MilAg-NP complex, miltefosine, and benznidazole was determined by calculating the fractional inhibitory concentration index (FICI) using the following formula with MPC values:

FICI = FIC_A_ + FIC_B_.

FIC_A_ = MPC of A in combination/MPC of A alone.

FIC_B_ = MPC of B in combination/MPC of B alone.

In this formula, A represents OA-MilAg-NP, and B represents benznidazole.

The calculated FIC index values were interpreted based on the following thresholds:

FIC index ≤ 0.5: synergy; FIC index = 0.50–0.75: partial synergy; FIC index = 0.75–1: additive; FIC index = 1.00–4.00: indifferent; FIC index ≥ 4.00: antagonism [16].

### Statistical Analysis

The antitrypanosomal activities of three different compounds (OA-MilAg-NP, miltefosine, and benznidazole) were compared based on percentage viability data. Data obtained at different time points (24, 48, and 72 h) and approximate dose levels (high, medium, low) were analyzed using one-way analysis of variance (ANOVA). In cases where a statistically significant difference was detected, Tukey's Honest Significant Difference (HSD) test was applied for post-hoc pairwise comparisons. All statistical analysis were performed using SPSS software (IBM SPSS Statistics, Version 22), and a p-value < 0.05 was considered statistically significant.

## Results

### FT-IR Characterization of the OA-MilAg-NP Complex

**Fig. 1:**
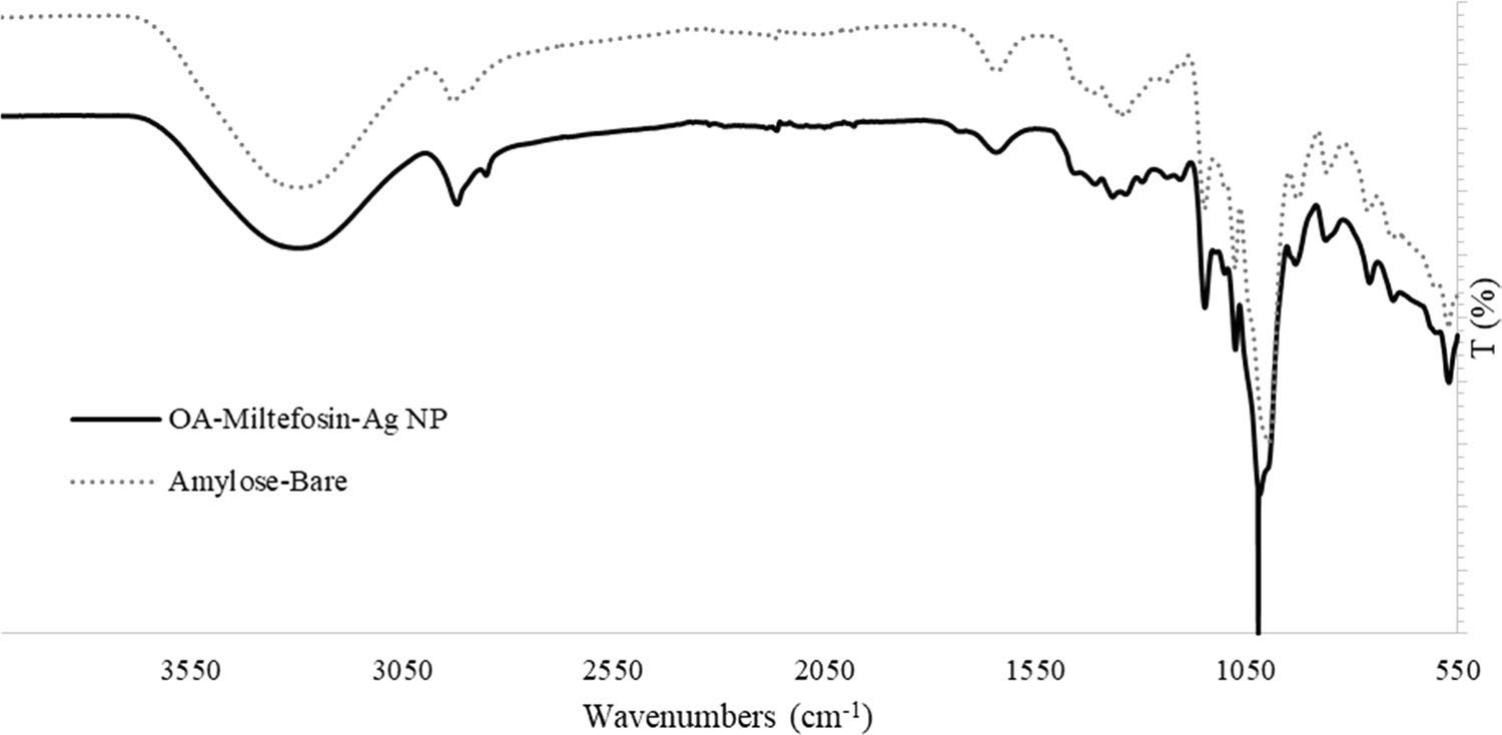
FT-IR spectrum of OA-MilAg-NP complex. The dashed line shows the characteristic peaks of oxidized amylose, which forms the core of the structure, while the black solid line represents the FT-IR spectrum of the OA-MilAg-NP complex. The differences in peaks between the two spectra indicate that miltefosine and silver have been successfully integrated into the structure

Characterization of the synthesized OA-MilAg-NP was achieved using FT-IR (Fourier transform infrared spectroscopy) analysis. Structural differences of OA-MilAg-NP and bare oxidized amylose were monitored using FT-IR spectra to prove synthesize of hybrid nanoparticle complex. Accordingly, the peaks at 1365 cm^−1^, 1296 cm^−1^, 1022 cm^−1^, 942 cm^−1^ and 611 cm-^1^ wavenumbers were newly appeared on OA-MilAg-NP spectrum. The peak observed at 1365 cm^−1^ was attributed to methyl group in phosphocholine structure of miltefosine. Additionally, the peaks 1296 cm^−1^ and 1022 cm^−1^ corresponds P = O and P–O–C stretching vibrations, respectively. The peak 942 cm^−1^ was associated with C-N stretching vibration. The vibration at 611 cm^−1^ may be attributed to P-O bending. Moreover, the peaks 2851 cm^−1^ and 2921 cm^−1^ which were sharper and clear on OA-MilAg-NP spectrum, corresponds the asymmetric and symmetric C-H stretching vibrations of the long alkyl chain of miltefosine. Those abovementioned results strongly emphasize that synthesize of hybrid OA-MilAg-NP complex was successfully performed. (Fig. 1)

### SEM and TEM Characterization of the OA–MilAg–NP Complex

**Fig. 2:**
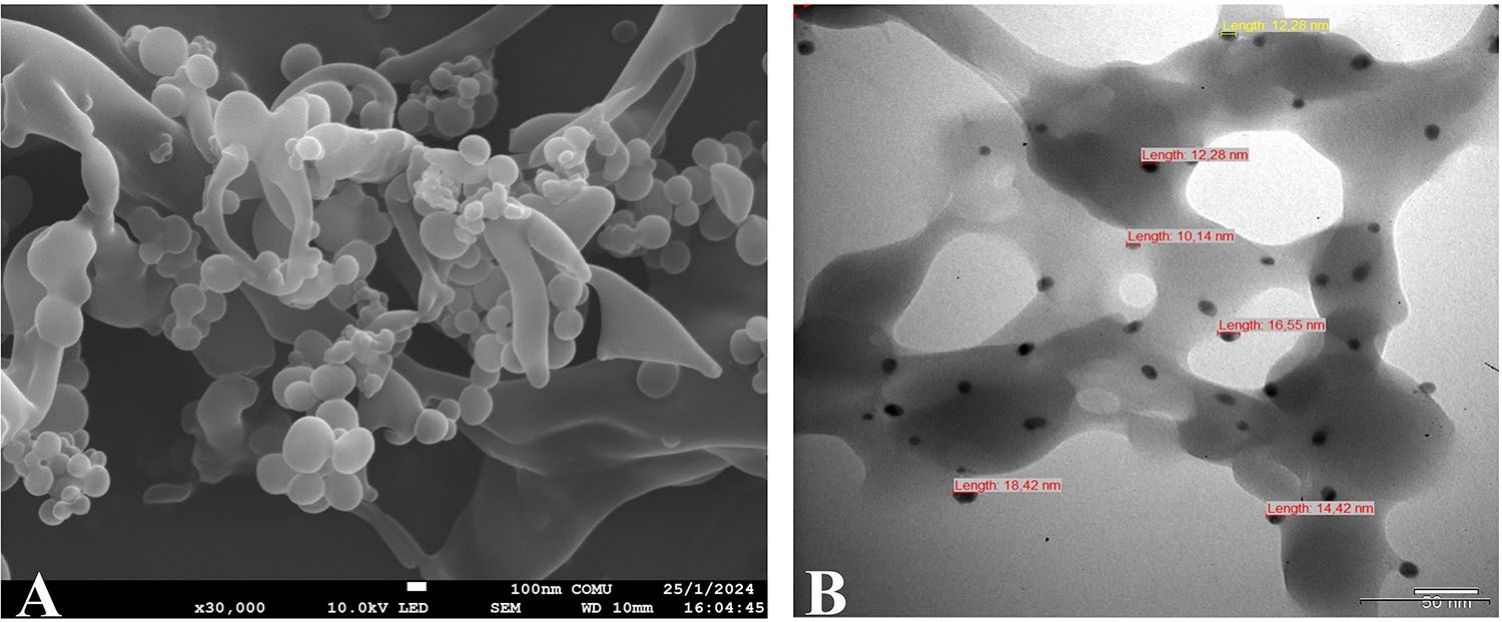
SEM (**A**) and TEM (**B**) images of the OA-MilAg-NP complex. A. The SEM image shows that the nanoparticles attached to the surface of oxidized amylose, which forms the core of the structure, have a round morphology and variable sizes. B. The TEM image reveals that the sizes of these surface-bound nanoparticles range from 10.14 to 18.42 nm

SEM analyses of the hybrid nanoparticle complex revealed that silver ions and miltefosine molecules were successfully incorporated into the surface of oxidized amylose, which forms the core of the structure, confirming the successful synthesis of the nanoparticle complex. The obtained images showed that the nanoparticles have a rounded morphological appearance, vary in size, and are successfully attached to the surface of oxidized amylose. TEM analysis revealed that the nanoparticle sizes ranged between 10.14 and 18.42 nm (Fig. 2)**.**

### Antitrypanosomal Activity Results of OA–MilAg–NP Complex, Miltefosine, and Benznidazole

**Fig. 3:**
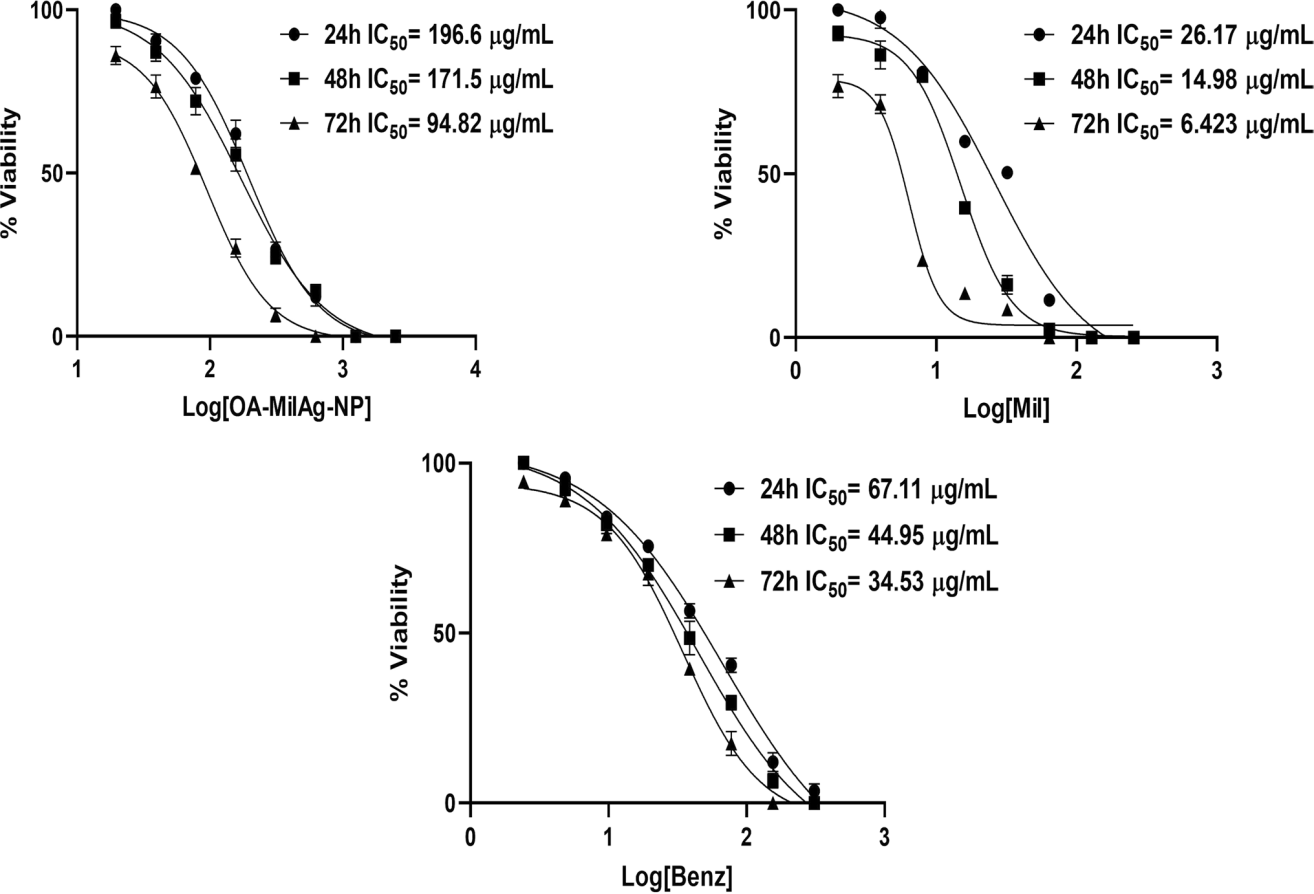
Graphs showing IC_50_ values ​​of OA-MilAg-NP complex, miltefosine and benznidazole against *T. cruzi* ATCC 50825 strain

A time-dependent decrease in the IC_50_ values of the OA-MilAg-NP complex, miltefosine, and benznidazole was observed, corresponding to an increase in antitrypanosomal activity (Table 1 and Fig. 3). While the MPC values, where all parasites were killed, remained constant at 24 and 48 h, the effective concentration decreased by one dilution at 72 h. It was determined that the IC_50_ and MPC values of benznidazole and miltefosine were close to each other and that they exhibited stronger antitrypanosomal activity compared to the OA-MilAg-NP complex. Miltefosine demonstrated significantly higher efficacy than the other two compounds at most time points and concentration levels (p < 0.05). Benznidazole was generally more effective than OA-MilAg-NP; however, no statistically significant difference was observed between benznidazole and miltefosine at certain time points, particularly at the highest concentration at 24 h (p = 0.3345). OA-MilAg-NP showed lower efficacy, especially at earlier time points, but its activity gradually increased over time. Nonetheless, in most pairwise comparisons, it remained significantly less effective than both miltefosine and benznidazole (e.g., miltefosine vs OA-MilAg-NP at 48 h, medium dose: p = 0.001). In conclusion, the findings indicate that miltefosine exhibited the most potent antiparasitic effect, followed by benznidazole, while OA-MilAg-NP demonstrated time-dependent but comparatively lower efficacy. All tested compounds were determined to have strong antitrypanosomal activity (Table 1 and Fig. 3).

**Table 1:** IC_50_ and MPC values ​​(µg/mL) of OA-MilAg-NP complex, miltefosine and benznidazole against *T. cruzi* ATCC 50825 strain

Active Ingredients	24 h		48 h		72 h	
	IC_50_	MPC	IC_50_	MPC	IC_50_	MPC
OA-MilAg-NP	196.6	625	171.5	625	94.82	312.5
Miltefosine	26.17	128	14.98	128	6.42	64
Benznidazole	67.11	155	44.95	155	34.53	77.5

*Mil* Miltefosine, *NP* Nanoparticle, *IC*_*50*_ 50% inhibitory concentration, *MPC* Minimum parasiticidal concentration.

### Cytotoxic Activity Results and Selectivity Index (SI) Values of OA-MilAg-NP Complex, Miltefosine, and Benznidazole

**Fig. 4:**
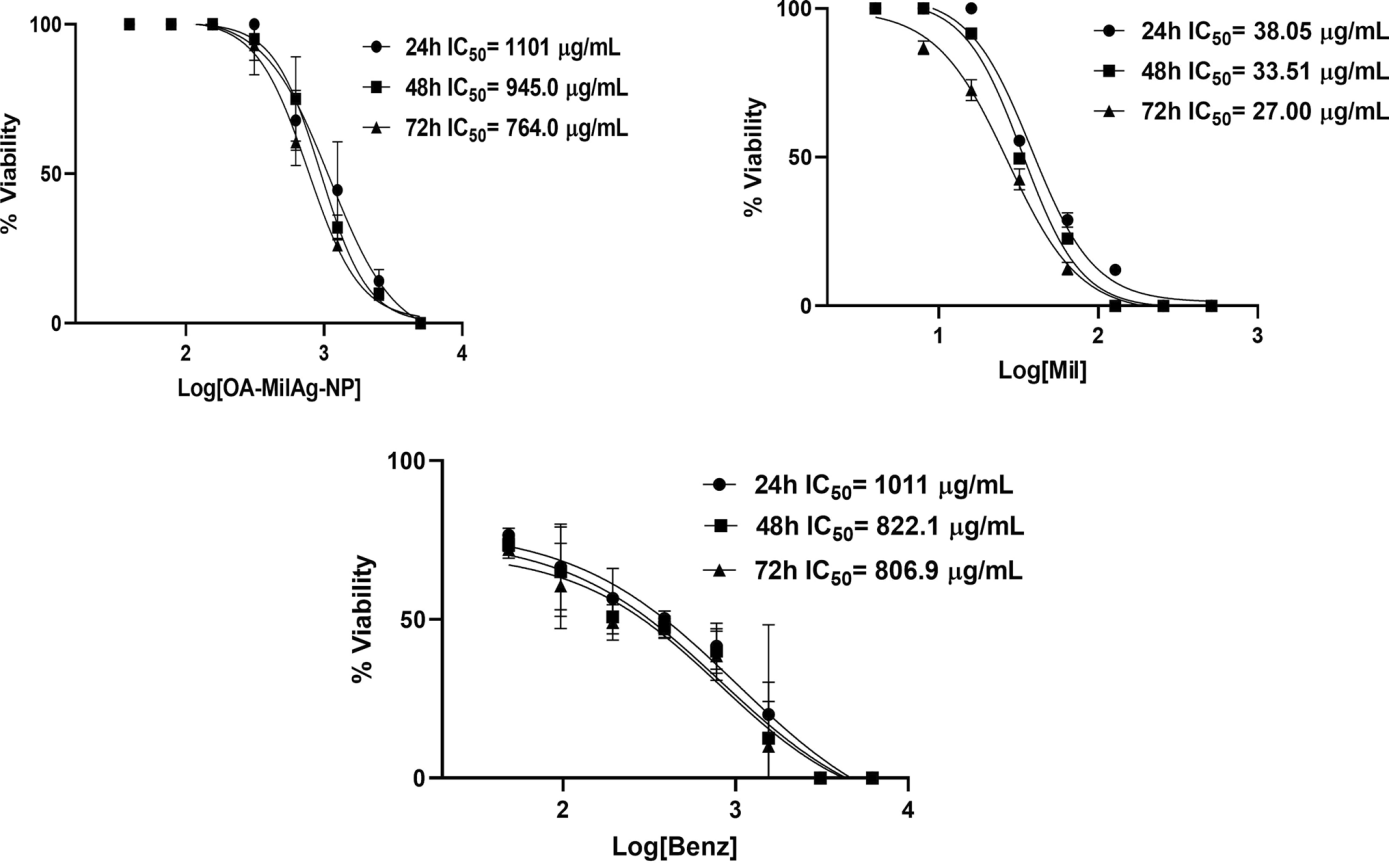
Graphs showing IC_50_ values ​​of OA-MilAg-NP complex, miltefosine and benznidazole against L929 Fibroblasts

The cytotoxic activities of the OA-MilAg-NP complex, miltefosine, and benznidazole against fibroblast cells were determined to range between 1101–764 µg/mL, 38.05–27 µg/mL, and 1011–806.9 µg/mL, respectively, at 24, 48, and 72 h. When SI values were calculated, miltefosine showed positive selectivity (SI > 1), whereas the OA-MilAg-NP complex and benznidazole exhibited high selectivity (Table 2 and Fig. 4). The antitrypanosomal activity values indicate that miltefosine is more effective than benznidazole. However, the cytotoxicity values also show that miltefosine is more toxic than benznidazole.

**Table 2:** Cytotoxicity and selectivity index values ​​(µg/mL) of OA-MilAg-NP complex, miltefosine and benznidazole against *T. cruzi* ATCC 50825 strain and L929 fibroblast cell line.

Active Ingredients	24 h (IC_50_)			48 h (IC_50_)			72 h (IC_50_)		
	*T.cruzi*	L929	SI	*T.cruzi*	L929	SI	*T.cruzi*	L929	SI
OA-MilAg-NP	196.6	1101	5.6	171.5	945	5.5	94.82	764	8
Miltefosine	26.17	38.05	1.5	14.98	33.51	2.2	6.42	27	4.2
Benznidazole	67.11	1011	15	44.95	822.1	18.2	34.53	806.9	23

*Mil* Miltefosine, *Nanoparticles IC*_*50*_% 50 inhibition concentration, *L929* Fibroblast cell line, *SI* Selectivity index

### Interactions Between OA-MilAg-NP Complex, Miltefosine, and Benznidazole

Synergistic interactions were observed for all incubation periods in the combinations of the OA-MilAg-NP complex and miltefosine with benznidazole (Table 3).

**Table 3:** Interaction of OA-MilAg-NP Complex and Miltefosine and benznidazole combinations against *T. cruzi* ATCC 50825 strain

Combinations	24 h		48 h		72 h	
	FICI	Interaction	FICI	Interaction	FICI	Interaction
OA-MilAgNP + Benz	0.499	Synergy	0.374	Synergy	0.312	Synergy
Mil + Benz	0.375	Synergy	0.375	Synergy	0.375	Synergy

*Ag* Silver, *NP* Nanoparticle, *Benz* Benznidazole, *Mil* Miltefosine, *FICI* Fractional inhibitory concentration index

**Fig. 5:**
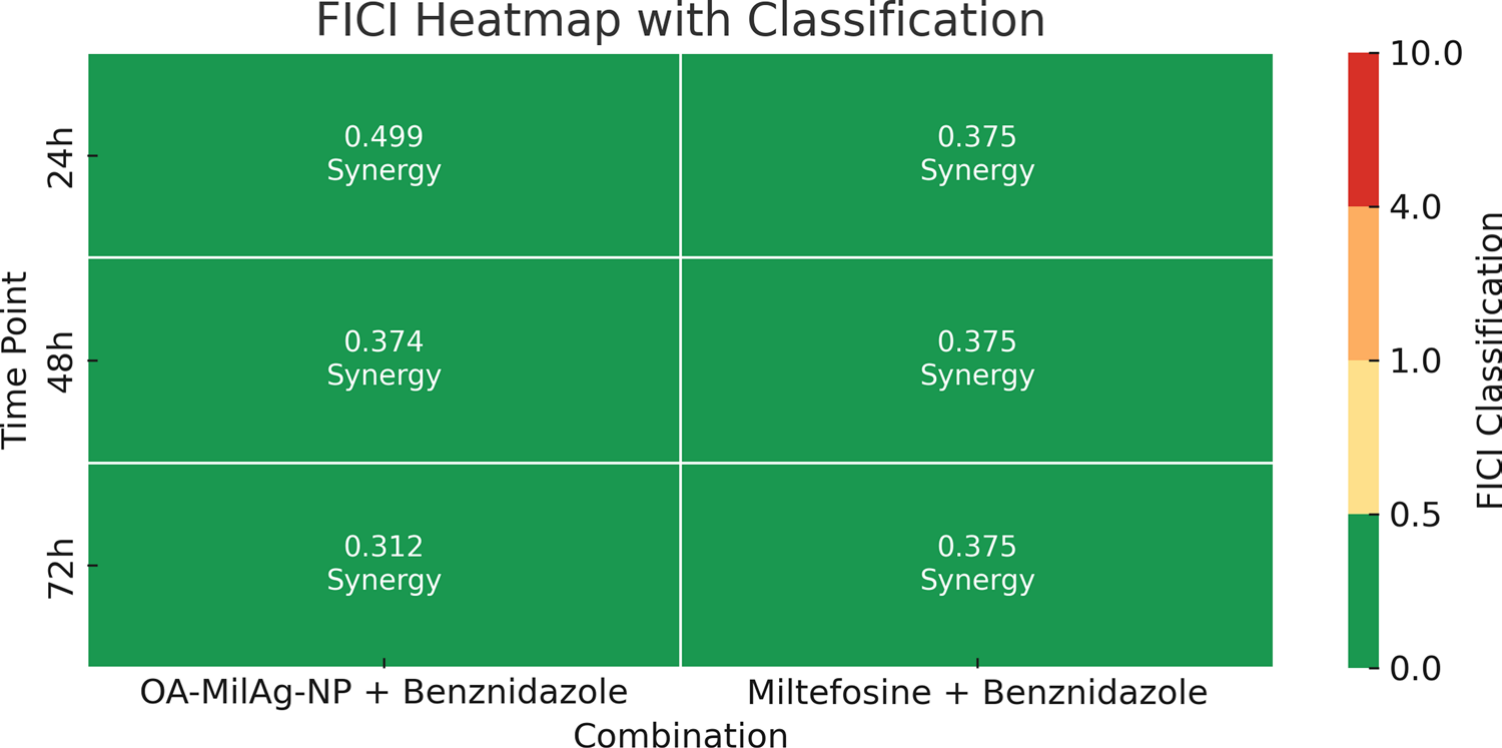
Heatmap showing the classified FICI values for the combinations of OA-MilAg-NP + benznidazole and miltefosine + benznidazole at 24, 48, and 72 h. All values fall within the synergy range (FICI ≤ 0.5)

FICI values were calculated for the combinations of OA-MilAg-NP + benznidazole and miltefosine + benznidazole at 24, 48, and 72 h. At all time points, the FICI values for both combinations were below 0.5. Specifically, the OA-MilAg-NP + benznidazole combination yielded FICI values of 0.499 at 24 h, 0.374 at 48 h, and 0.312 at 72 h. The Miltefosine benznidazole combination showed a constant FICI value of 0.375 across all time points. According to standard classification, all values were within the synergy range (FICI ≤ 0.5) (Fig. 5).

**Fig. 6:**
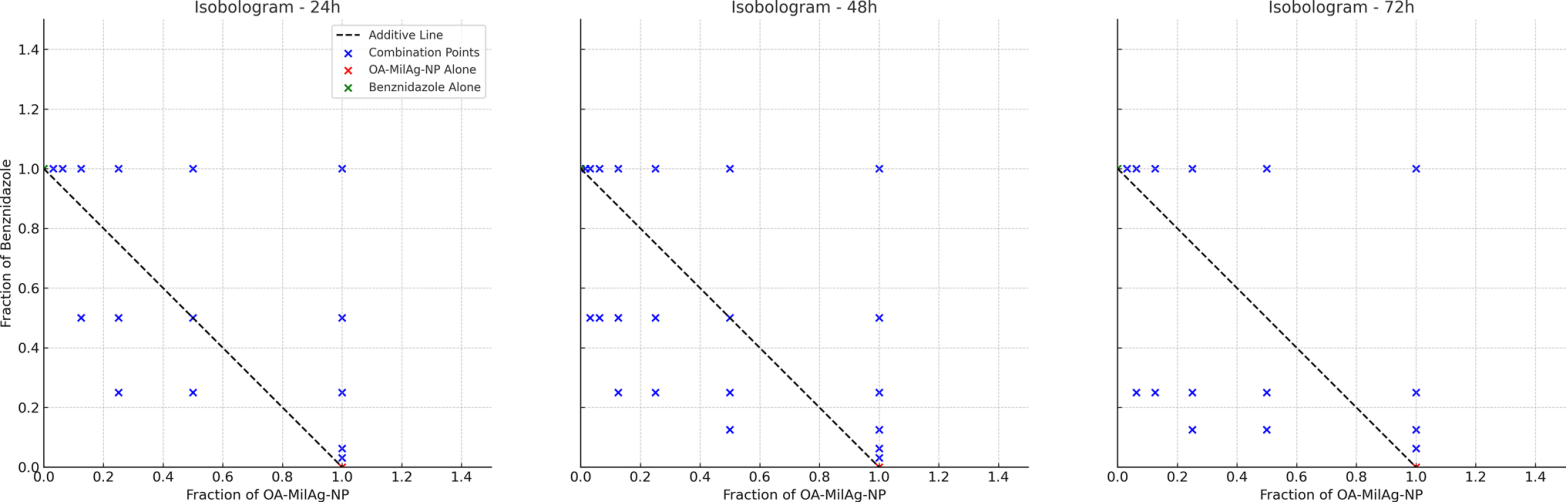
Isobolograms representing the interaction between OA-MilAg-NP and benznidazole at 24, 48, and 72 h. Data points represent combinations that achieved complete parasite elimination, plotted as fractional concentrations normalized to the minimum fully effective monotherapy doses

**Fig. 7:**
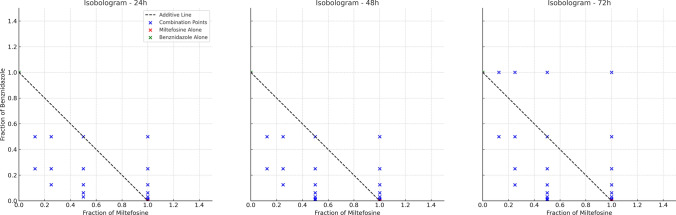
Isobolograms representing the interaction between miltefosine and benznidazoleat 24, 48, and 72 h. Data points represent combinations that achieved complete parasite elimination, plotted as fractional concentrations normalized to the minimum fully effective monotherapy doses

Isobologram analyses were conducted at 24, 48, and 72 h to evaluate the interactions between the combinations of miltefosine + benznidazole and OA-MilAg-NP + benznidazole. Only combinations that resulted in complete parasite elimination (100% inhibition) were considered. For each combination, the fractional concentrations of both compounds were normalized to the minimum monotherapy doses that produced full inhibitory effects, and the data were plotted accordingly. The miltefosine + benznidazole combination consistently demonstrated a synergistic interaction across all time points, as most of the data points were located below the line of additivity. This effect was most pronounced at 48 h, where significantly lower fractional concentrations of both drugs achieved complete inhibition. Although the OA-MilAg-NP + benznidazole combination included fewer fully effective data points, it exhibited a similar trend toward synergy, particularly at 72 h, where several points fell below the additivity line. These findings suggest that both combinations have synergistic potential and may enhance antitrypanosomal efficacy when co-administere (Fig. 6 and 7).

## Discussion

Chagas disease, caused by *Trypanosoma cruzi*, is considered a neglected tropical disease due to the lack of safe and effective treatments. Current treatment options are limited to nifurtimox and benznidazole, both of which were developed over 40 years ago [17]. While etiological treatment in the acute phase leads to high rates of parasite clearance and seronegative results, treatment success in the chronic phase remains debatable. Adverse events, particularly in adults, are common and often lead to discontinuation of treatment [18]. Pharmaceutical companies have shifted their focus to long-term use drugs rather than investing in costly R&D for new molecule development [19]. This situation underscores the need for strategies aimed at discovering new antitrypanosomal drugs.

Drug repurposing is the process of using an existing drug or drug candidate for a new therapeutic indication or medical condition not originally intended. In this process, the unintended side effects of drug molecules can serve as markers for discovering their effectiveness in entirely different medical conditions. Typically, drugs with proven safety in humans are tested and developed for efficacy in diseases other than the one for which they were originally designed. This process bypasses early drug development stages, thereby reducing risks and costs [20]. However, like traditional drugs, repurposed drugs face challenges related to formulation and delivery, such as poor aqueous solubility, limited stability, and/or incompatibility with new delivery routes. Nanotechnology plays a significant role in promoting the efficient use of highly active but poorly delivered drugs. It is frequently explored as a means to overcome challenges such as poor solubility, toxicity, and multidrug resistance in drug delivery systems [21].

Miltefosine is considered a protein kinase B (PKB) inhibitor, a key regulator of intracellular signaling essential for cell viability [22]. However, the mechanisms underlying its antiprotozoal activity remain incompletely understood. Some evidence suggests that it inhibits phosphatidylcholine and sphingomyelin synthesis, induces apoptosis [23], or inhibits mitochondrial cytochrome c oxidase and alters the organelle membrane [22]. Similarly, it has been proposed that miltefosine inhibits parasite phosphatidylcholine biosynthesis through the transmethylation pathway [24]. Recently, it has been suggested that miltefosine targets a sphingosine-activated plasma membrane Ca^2^⁺ channel in both *Leishmania donovani* and *T. cruzi*, affecting intracellular calcium homeostasis [25].

Several studies in the literature demonstrate the efficacy of miltefosine against *T. cruzi* in both in vitro and in vivo [17, 26, 27]. A recent study by Gulin et al. (2022) reported IC_50_ values of miltefosine against *T. cruzi* amastigote and trypomastigote forms as 0.48 µM and 0.55 µM, respectively, and LC_50_ (lethal concentration) values as 29.56 µM and 32.87 µM, respectively. They also found that the combination of miltefosine and benznidazole exhibited synergistic and additive interactions against trypomastigotes and amastigotes, respectively. Additionally, they reported that the combination was more effective than miltefosine alone in reducing parasitemia in an in vivo rodent model [17].

In our study, unlike those in the literature, we synthesized a hybrid oxidized amylose miltefosine silver nanoparticle (OA-MilAg-NP) complex by combining miltefosine with silver and compared its efficacy against *T. cruzi* ATCC 50825 strain with that of miltefosine and benznidazole. We also investigated the in vitro interactions of both miltefosine and the OA-MilAg-NP complex with benznidazole. In our previous study, we demonstrated that a natural active component (curcumin) and silver NP complex exhibited strong antileishmanial activity with low cytotoxicity against *Leishmania* species [7]. Based on this, we aimed to enhance the efficacy of miltefosine while reducing its toxicity against *T. cruzi*, a parasite closely related to *Leishmania* species, using nanoparticle formulations and to identify synergistic interactions in combination with benznidazole.

The synthesized miltefosine-silver NP complex was prepared using a method involving oxidized amylose as the core and miltefosine and silver ions at the nanoparticle level on the surface, as in our previous study [5, 9, 10]. FT-IR spectra of the OA-MilAg-NP complex revealed peak shifts and splitting, indicating the successful attachment of functional groups to the oxidized amylose surface. SEM and TEM imaging showed that the OA-MilAg-NP complex had a round morphology, variable sizes, and a size range of 10.14–18.42 nm.

A recent study by Latifi et al. (2024) synthesized miltefosine as chitosan nanoparticles with an average size of 46.61 nm and reported that the formulation was more effective against *Acanthamoeba* trophozoites and cysts than miltefosine alone. The same study also indicated that the NP formulation exhibited significantly lower cytotoxicity on Vero cell lines compared to miltefosine [28].

In our study, we compared the toxicity of the OA–MilAg–NP complex to L929 mouse fibroblasts with that of miltefosine and benznidazole and calculated the selectivity index (SI) for each compound. Benznidazole, as a conventional drug, exhibited the highest SI values during 24, 48, and 72-h incubation periods. Notably, the OA-MilAg-NP complex showed at least twice the selectivity of miltefosine alone based on SI values (Table 2). Although miltefosine alone appeared to be more effective against *T. cruzi* than the OA-MilAg-NP complex, it exhibited higher cytotoxicity. However, the SI value of the hybrid complex was found to be higher than that of miltefosine alone. A possible reason for this may be the low release of miltefosine from the complex.

A study investigating the efficacy of miltefosine formulated with lipid NPs against cutaneous leishmaniasis reported that lipid NPs had an average size of 160.8 nm, six times lower cytotoxicity against macrophage cell lines, and strong antileishmanial activity in vivo [29]. We believe that the much smaller NP size (10.14–18.42 nm) achieved with our synthesis method compared to other studies [30] may be a significant factor in enhancing antitrypanosomal activity.

In recent years, various biopolymer- and lipid-based nanoparticle systems have gained attention in drug delivery due to their biological advantages. Among these, oxidized amylose-based nanoparticles stand out for their high biocompatibility, enzymatic degradability, and suitability for surface functionalization. Compared to commonly used systems such as chitosan and liposomal nanoparticles, oxidized amylose nanoparticles offer low cytotoxicity and controlled drug release, making them a suitable option for long-term drug administration. While chitosan can enhance cellular uptake, certain forms may exhibit cytotoxicity; liposomes, although effective in drug transport, have limitations in terms of stability. Considering these factors, oxidized amylose nanoparticles can be regarded as a balanced and effective drug carrier system. [10, 31].

There are limited studies in the literature on the combination of benznidazole and miltefosine against *T. cruzi.* Gulin et al. (2022) noted that the synergistic interaction of benznidazole and miltefosine observed in vitro was also effective in vivo [17]. In our study, a synergistic interaction was observed between miltefosine and benznidazole. Furthermore, unlike the literature, our study demonstrated a synergistic interaction between the OA–MilAg–NP complex and benznidazole. The isobologram findings indicate that both miltefosine + benznidazole and OA–MilAg–NP + benznidazole combinations possess synergistic potential against the target parasite. The miltefosine combination demonstrated a more consistent and pronounced synergy, particularly at 48 h, whereas the OA-MilAg-NP combination showed a more limited but still notable synergistic trend at later time points. These results suggest that such combinations may offer enhanced therapeutic efficacy and justify further investigation, particularly in efforts to reduce individual drug dosages and associated toxicity. The data we obtained represent the first evidence demonstrating the hybrid synthesis of the OA-MilAg-NP complex based on oxidized amylose and its efficacy against *T. cruzi.*

The data obtained on the efficacy of our oxidized amylose-based OA-MilAg-NP complex against *T. cruzi* and its interaction with benznidazole represent a novel contribution to the literature and provide valuable insights for the treatment of Chagas disease.

## Limitations

The limitation of the study is that certain characterization methods for the hybrid NP complexes—such as hydrodynamic size, polydispersity index (PDI), zeta potential, and loading efficiency (LE)—could not be performed.The another limitation of this study is that the antitrypanosomal activity of the hybrid NP complex and miltefosine was evaluated only in vitro and exclusively on epimastigote forms. Valuable data obtained from intracellular amastigotes and in vivo animal models could provide a more comprehensive understanding of the overall picture. Our ongoing studies are being planned accordingly.

## Conclusion

Investigating and developing new bioactive compounds with lower toxicity compared to conventional drugs for Chagas disease treatment through nanotechnology applications can provide significant contributions to therapeutic efficacy in the literature. The hybridization of miltefosine with silver nanoparticles, which has demonstrated efficacy against leishmaniasis, and the strong antitrypanosomal activities of these novel molecules along with their synergistic interactions with existing antitrypanosomal drugs, can address significant gaps in the literature. These findings pave the way for designing new drug combinations, reducing side effects, and preventing resistance development, offering valuable insights for future studies.

## Data Availability

No datasets were generated or analysed during the current study.
